# Role of the immune system in liver transplantation and its implications for therapeutic interventions

**DOI:** 10.1002/mco2.444

**Published:** 2023-12-13

**Authors:** Guanrong Chen, Xin Hu, Yingchen Huang, Xiaonan Xiang, Sheng Pan, Ronggao Chen, Xiao Xu

**Affiliations:** ^1^ The Fourth School of Clinical Medicine Zhejiang Chinese Medical University Hangzhou China; ^2^ Zhejiang University School of Medicine Hangzhou China; ^3^ Key Laboratory of Integrated Oncology and Intelligent Medicine of Zhejiang Province Hangzhou China; ^4^ Department of Hepatobiliary and Pancreatic Surgery The First Affiliated Hospital Zhejiang University School of Medicine Hangzhou China; ^5^ Zhejiang Chinese Medical University Hangzhou China

**Keywords:** cell therapy, complication, immune system, ischemia–reperfusion injury, liver transplantation

## Abstract

Liver transplantation (LT) stands as the gold standard for treating end‐stage liver disease and hepatocellular carcinoma, yet postoperative complications continue to impact survival rates. The liver's unique immune system, governed by a microenvironment of diverse immune cells, is disrupted during processes like ischemia–reperfusion injury posttransplantation, leading to immune imbalance, inflammation, and subsequent complications. In the posttransplantation period, immune cells within the liver collaboratively foster a tolerant environment, crucial for immune tolerance and liver regeneration. While clinical trials exploring cell therapy for LT complications exist, a comprehensive summary is lacking. This review provides an insight into the intricacies of the liver's immune microenvironment, with a specific focus on macrophages and T cells as primary immune players. Delving into the immunological dynamics at different stages of LT, we explore the disruptions after LT and subsequent immune responses. Focusing on immune cell targeting for treating liver transplant complications, we provide a comprehensive summary of ongoing clinical trials in this domain, especially cell therapies. Furthermore, we offer innovative treatment strategies that leverage the opportunities and prospects identified in the therapeutic landscape. This review seeks to advance our understanding of LT immunology and steer the development of precise therapies for postoperative complications.

## Graphical abstract

The liver is rich in immune cells, including innate immune cells and adaptive immune cells, which constitute the immune microenvironment. The immune microenvironment of liver may change in the ischemia–reperfusion stage and the acute rejection stage after liver transplantation and cause complications. In this process, Kupffer cells as the main innate immune cells and T cells as the main adaptive immune cells participate in the reaction.

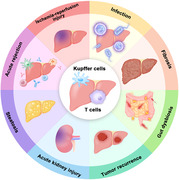

## 1 INTRODUCTION

Liver transplantation (LT) is invariably considered the best treatment for end‐stage liver disease and hepatocellular carcinoma.^1^, ^2^ Disruption of the immune microenvironment after transplantation leads to acute rejection (AR), ischemia–reperfusion injury (IRI), and other processes that affect the success rate of transplantation and patient survival.^3^, ^4^ To improve prognosis, clinical immunosuppressive agents such as glucocorticoids and calcineurin inhibitors (CNIs) have been widely used.^5^ Nevertheless, immune imbalance and secondary complications resulting from immunosuppressive agents can lead to subsequent effects.^6^, ^7^ Hence, a thorough analysis of alterations in the immune microenvironment and immune cell dynamics following LT is imperative.

Several decades ago, it became evident that the liver functions as a sophisticated immune organ, teeming with a diverse array of both innate and adaptive immune cells.^8^, ^9^ These immune cells promote the formation of an immunological microenvironment in the liver through cytokine‐mediated interactions, which have been associated with various liver diseases.^10^, ^11^ In the perspective of LT, greater consideration should be given to these alterations in the immune microenvironment. In 1985, needle biopsies of several patients experiencing AR after LT revealed the presence of biliary multinucleate cells and lymphocyte infiltration.^12^ These findings implied that immune cell infiltration and disruption of the immune microenvironment in LT could contribute to the reduced posttransplantation survival rate. As research into the liver's immune microenvironment advances, drugs targeting the regulation of T cells, particularly Sirolimus, have shown promise in maintaining LT tolerance.^13^ Nonetheless, caution is still necessary due to the potential risks of withdrawal rejection and additional organ damage associated with drug usage.^14^ Therefore, there is an urgent need to develop therapies targeting immune microenvironment induction to mitigate post‐LT damage.

**FIGURE 1:**
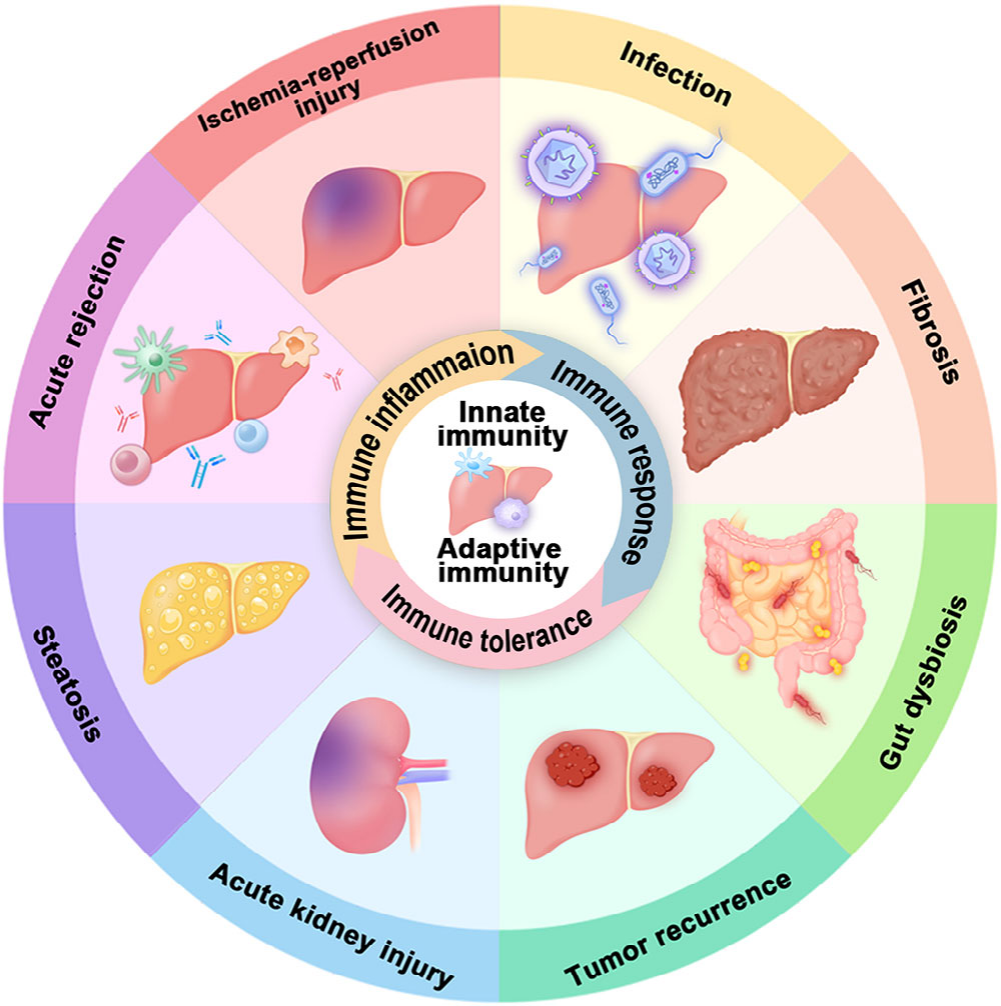
The role of immune cells in liver transplantation. The immune system of the liver is mainly composed of innate immunity and adaptive immunity. After liver transplantation, the liver immune system undergoes immune inflammation and immune damage, leading to subsequent complications. On the other hand, the liver immune system subsequently maintains immune tolerance. Based on the role of the immune system in liver transplantation can be used as an entry point to treat complications.

Within the liver, there exists a subset of immune cells with a tolerant profile, comprising macrophages, dendritic cells (DCs), and regulatory T cells (Tregs).^15^ To overcome the constraints of immune responses in LT drug therapy, cell therapy has emerged as a novel approach for immune regulation.^16^ It provides new insights and approaches for potential future therapies involving immune cell populations. In this review, we center on the composition of the immune microenvironment within the liver, primarily governed by immune cells, and its correlation with complications arising from LT. First, we introduce the categorization of the liver's immune system and the makeup of its immune microenvironment. Subsequently, we provide an overview of the roles of innate and adaptive immunity at various stages following LT, encompassing IRI and AR. We have compiled the current cellular therapy approaches for innate and adaptive immune cells in LT based on the immune mechanisms involved. Following that, we also summarized alterations in the immune microenvironment and other complications, such as acute kidney injury (AKI), intestinal flora dysregulation, and liver cancer recurrence. Finally, we provide a summary of the aforementioned topics and offer insights into future prospects. Throughout our discussion, we focused on macrophages and T cells as the primary subjects to elucidate their immune effects (Figure 1).

## 2 HEPATIC IMMUNE SYSTEM AND IMMUNE MICROENVIRONMENT

**FIGURE 2:**
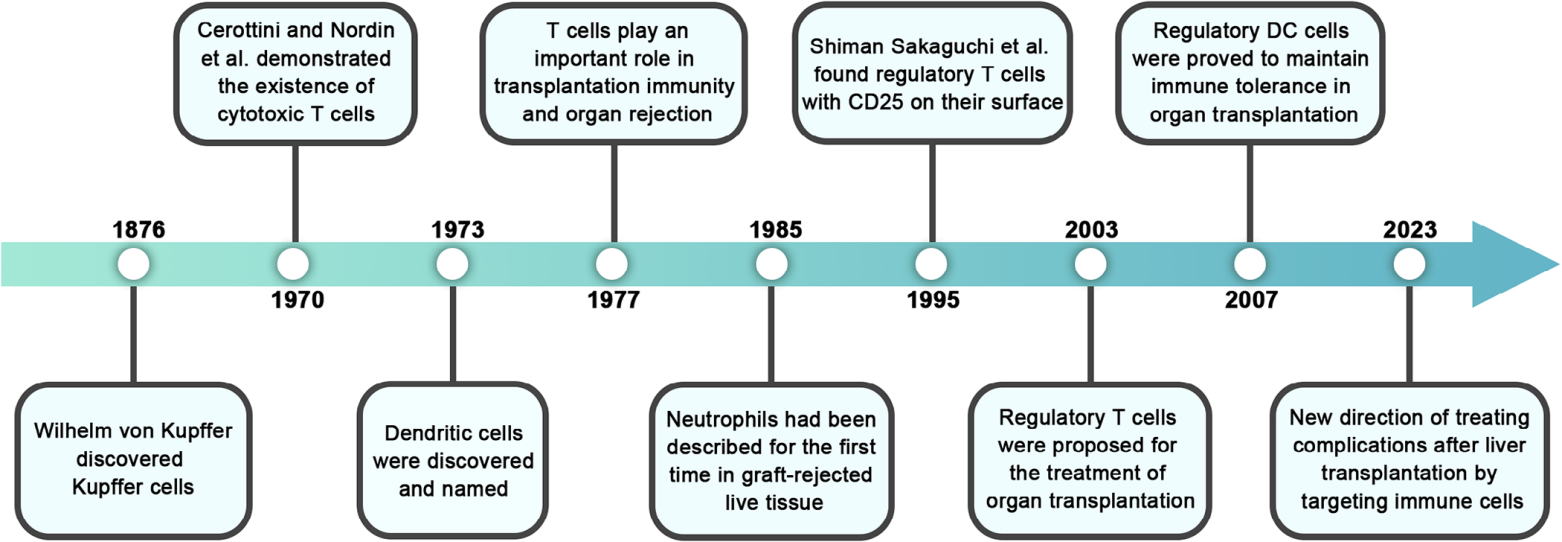
Timeline of important events in liver immune cell discovery and development. In the early 19th century, Kupffer cells was the first detected immune cell in the liver. In the 20th century, immune cells such as T‐cells and dendritic cells and other immune cells were gradually discovered and confirmed by organ transplants in the liver transplantation. In addition to causing organ exclusion, immune resistant cells, which represent the regulated type T cells and the regulated dendritic cells, begin to be the focus of organ transplantation in the context of the immune cell phenotype. Nowadays, the treatment of liver transplantation complications by targeting immune cells, especially tolerant immune cells, has become a hot topic.

The liver, a complex immune organ in the human body, harbors a plethora of immune cells that play an indispensable role in both the innate and adaptive branches of the immune system. Within these immune cell populations, recognized constituents of the innate immune system in the liver encompass liver resident macrophages, also known as Kupffer cells (KCs), DCs, natural killer (NK) cells, as well as other elements such as invariant NKT (iNKT) cells, and more.^17^, ^18^ Beyond their roles in immune response and the maintenance of immune tolerance, KCs and DCs collaborate with appropriate immune cells to orchestrate effective immune responses.^19^, ^20^ In the liver, innate immune cells, notably KCs, coordinate with adaptive immune cells, which include Tregs, helper T (Th) cells, B cells, and plasma cells, to orchestrate subsequent immune and inflammatory responses in liver diseases.^21^, ^22^, ^23^ In addition to the contributions of immune cells, inflammatory mediators like interleukin (IL)‐12 play a pivotal role in shaping the immune microenvironment.^24^ The intricate interplay of immune cells and molecules within the microenvironment undergoes alterations and dynamic interactions in the contexts of LT and liver diseases (Figure 2).

### 2.1 Innate immunity

#### 2.1.1 Macrophages

Hepatic macrophages are primarily categorized into two main groups: KCs and monocyte‐derived macrophages (MoMϕs).^25^ KCs, residing in the liver, are recognized as the predominant component of liver macrophages. They were initially discovered in the liver by Karl Wilhelm von Kupffer in 1876, and he named them after himself.^26^ Following this groundbreaking discovery, researchers also turned their focus toward this phagocytic inflammatory cell. KCs play a crucial role in preserving physiological homeostasis by upholding immune tolerance and facilitating the clearance of iron and intestinal microbial products.^27^ In the context of activation, liver macrophages make substantial contributions to processes such as hemolysis and metabolism, even in the presence of liver diseases.^28^, ^29^, ^30^ KCs are typically derived from erythro‐myeloid progenitor cells and can proliferate, whereas MoMϕs are primarily replenished by peripheral blood mononuclear cells in adulthood.^31^, ^32^ As immune cells, KCs exhibit a multifaceted relationship with other immune cells. Siwicki et al. employed anti‐cluster of differentiation (CD)40 treatment in mice as a model for Th1‐mediated immunotherapy. They substantiated that KCs have the ability to detect interferon (IFN)‐γ derived from lymphocytes, subsequently leading to a robust neutrophil response.^33^ In general, KCs are regarded as immune‐tolerant macrophages, whereas MoMϕs are classified as inflammatory cells.^34^ During pathological conditions such as liver injury, infections, and tumor development, KCs undergo polarization and actively recruit monocytes from the bloodstream.

In early mouse studies, KCs and MoMϕs were differentiated based on distinct cell surface markers. MoMϕs are categorized into two groups: LY6C^+^ monocytes characterized by CCR2^+^CX3CR1^+^CD43^−^ and LY6C^−^ monocytes displaying CX3CR1^hi^CCR2^−^CD43^+^ phenotypes.^35^ KCs, on the other hand, were confirmed to express CD11b^low^, F4/80^hi^, CD68^+^, CX3CR1^−^, and Clec4F^+^.^36^, ^37^ Through the use of single‐cell sequencing, researchers have gained deeper insights into the diversity of macrophages.^38^ CD68^+^MARCO^−^ macrophages promote the recruitment of inflammatory cells, whereas CD68^+^MARCO^+^ KCs play a role in maintaining immune tolerance and dampening inflammation. They achieve this through the expression of various transcription factors, including VSIG4, CD163, HMOX1, and C1QC.^39^, ^40^ Thanks to advancements in detection techniques, researchers have delved deeper into the diversity of liver macrophages. At present, macrophage phenotypes are categorized as either proinflammatory M1, induced by lipopolysaccharide (LPS) and IFN‐γ, or anti‐inflammatory M2, activated by IL‐4 and IL‐13.^41^, ^42^ Nevertheless, due to the intricate phenotypic nature of liver macrophages, this classification is an oversimplification.^43^ Recent studies have brought regulatory macrophages (Mregs) into focus in the field of organ transplantation due to their mechanisms for promoting immune tolerance and suppressing inflammation.^44^ Mregs, distinguished by the presence of CD14^+^, CD206^+^, CD163^+^, ABCB5, and DCSTAMP biomarkers, primarily play a role in IL‐10‐mediated inflammatory responses,^45^, ^46^, ^47^ To date, research on Mregs in the context of LT remains limited, suggesting a potential avenue for future research in the field of transplantation immune regulation. Special subpopulations of macrophages also exist in the peritoneal cavity outside the liver. Large peritoneal macrophages are characterized by the expression of F4/80^hi^ and GATA6^+^, as well as CD11b^hi^, accounting for approximately 90% of these cells. In contrast, small peritoneal macrophages, are defined by F4/80^low^ and CD11b^low^.^48^, ^49^ While research on peritoneal macrophages remains limited, some studies have indicated their preferential recruitment during liver injury.^50^ Macrophages hold a pivotal position within the liver's innate immune system, serving both to trigger inflammation and to maintain immune tolerance.

#### 2.1.2 DCs

DCs were officially characterized in peripheral lymphoid tissues in 1973.^51^ DCs express the major histocompatibility complex (MHC) molecule, human leukocyte antigen (HLA)‐DR, and the integrin CD11c on their surface. Subspecies classification is based on the expression of specific markers, including BDCA‐1 (CD1c), BDCA‐2 (CD303), BDCA‐3 (CD141), and BDCA‐4 (CD304).^52^, ^53^ In humans, DCs can be categorized into two groups of conventional myeloid DCs (cDCs): CD141^+^ CD14^−^DCs, referred to as cDC1 cells, and CD1c^+^ CD14^−^DCs, known as cDC2 cells. Additionally, there is a third category consisting of CD303^+^ CD304^+^ plasmacytoid DCs (pDCs).^53^, ^54^ cDC2 cells represent the predominant population of DCs in the human liver. They play a role in activating Th2 and Th17 responses and can also secrete IL‐10 to induce a subdued immune response in T cells.^55^ In contrast to cDC2, cDC1 engages with CD8^+^ T cells via surface Toll‐like receptors 2 (TLR2) and Toll‐like receptors 4 (TLR4), leading to the initiation of adaptive immunity.^56^ When compared with the two types of cDCs interacting with T cells, pDCs exhibit a broader range of immune interactions. Research has demonstrated that pDCs decrease in the context of chronic viral infections, making them a valuable indicator of drug efficacy.^57^ Through the activation of B cells, NK cells, and T cells via type‐1 IFN, pDCs promptly exert antiviral and immunostimulatory effects.^58^ Conversely, IFN‐α produced by pDCs can stimulate hepatocytes to express CX3CL1, potentially leading to the recurrence of hepatocellular carcinoma.^59^ In addition to these three major subtypes, there are monocyte‐derived DCs (moDCs) obtained in vitro and regulatory DCs (DCregs), which are recognized as key players in immune tolerance regulation in LT.^60^

#### 2.1.3 NK cells

NK cells are integral components of the innate immune system. The nonphagocytic nature of liver NK cells was initially identified, and they were coined as “pit cells” in 1976.^61^ Hepatic NK cells can be categorized into two primary types based on their surface markers: circulating NK (c‐NK) cells and liver‐resident NK (lr‐NK) cells.^62^ In human NK cells, the classification of the two types is better achieved using CD56^dim^ and CD56^bright^. Among these, Ir‐NK cells have been observed to express CD49a^+^CD56^bright^.^63^, ^64^ Traditionally, NK cells eliminate cells by detecting low levels of MHC‐I expression, which is common in infected cells and tumors. NK cells maintain a balance between activating receptors like CD16, CD94, NK cell group 2D (NKG2D), NKp46, NKp44, NKp30, CD226, and NK granulo7, and inhibitory receptors such as CD96, TIGIT, LAG3, and killer cell immunoglobulin receptors (KIR).^65^ When it comes to interacting with immune cells, Ir‐NK cells promote T cell apoptosis by increasing the expression of ligands such as tumor necrosis factor (TNF)‐related apoptosis‐inducing ligand and NKG2D in response to elevated IFN‐α levels.^66^ NK cells can further enhance the anti‐tumor therapeutic effect when combined with DC following CD47 inhibition.^67^

#### 2.1.4 Other innate immune cells

The liver's innate immune cell repertoire encompasses neutrophils, iNKT cells, mucosal‐associated invariant T (MAIT) cells, γδT cells, and innate lymphoid cells (ILCs).^68^, ^69^, ^70^, ^71^ Neutrophils serve a diverse array of functions, which include triggering early inflammatory responses, phagocyte fragmentation, inducing macrophage phenotypic changes, and facilitating vascular regeneration.^72^, ^73^, ^74^, ^75^ iNKT cells are recognized for their roles in inflammation, tumorigenesis, and allergic reactions.^76^ Another subtype, type II NKT cells, supports tissue repair.^77^ ILCs can influence adaptive immunity and hinder CD8^+^ T cell responses through IL‐2 mediation.^78^ Meanwhile, MAIT and γδT cells, although contributing to their innate immune responses, can also trigger metabolism‐related diseases via the enterohepatic axis.^79^, ^80^

### 2.2 Adaptive immunity

#### 2.2.1 T cells

The healthy liver contains a substantial population of T lymphocytes responsible for mediating cellular immunity. Among these, αβTCR‐expressing T cells are the primary agents of adaptive immunity. They constitute approximately 95% of the total human peripheral blood T cell population, in contrast to the γδT cells mentioned earlier.^81^ Adaptive immune T cells in the liver, which coexpress CD3 on their surface, can be categorized into CD8^+^ T cells, CD4^+^ T cells, Tregs, and Th cells. They establish immune connections through the T cell receptor (TCR).^82^, ^83^ In regular conditions, immune tolerance is upheld through the recognition of MHC molecules between T cells and antigen‐presenting cells (APCs), such as DCs and KCs.^84^ During pathological conditions, APCs can provoke additional immune responses by modifying the production of MHC molecules.

CD4^+^ T cells typically act as preferred activators of cellular immunity by enhancing MHC‐II interactions on their cell surface, especially in an inflammatory environment.^85^, ^86^ Further studies have unveiled that within CD4^+^ T cells classified based on CD69 expression, CD69^HI^ CD4^+^ T cells are identified as liver‐resident CD4^+^ T cells. In contrast, CD69^INT^ CD4^+^ T cells expressing a chemokine receptor profile of CX3CR1^+^, CXCR3^+^, and CXCR1^+^ exhibit a more diverse phenotype.^87^ In the realm of APCs, type‐2 DCs (cDC2) play a significant role in the interaction with CD4^+^ T cells and may hold clinical importance in targeting immune checkpoints.^88^ In any case, CD4^+^ T cells have a strong connection with APCs and are vital for immune regulation. These CD4^+^ T cells produce cytokines and serve as supporting players in the immune response. Helper T cells, known as Th cells, can be categorized into different types, including Th1, Th2, Th17, and Th22.^83^, ^89^, ^90^ Within these, Th1 cells release IFN‐γ, IL‐2, TNF‐α, IL‐6, IL‐8, and IL‐1β, which trigger cell‐mediated immune responses. On the other hand, Th2 cells produce IL‐1ra, IL‐4, IL‐5, and IL‐10, promoting humoral immunity.^91^, ^92^ Th17 cells exhibit dual plasticity, with the ability to serve both pathogenic and protective roles in host immune function. IL‐17 and IL‐22 play a significant role in the initiation and progression of liver cancer, as well as acute and chronic liver injuries.^93^ Given the flexibility of Th17 cells, the regulation of Th17 type conversion has become a central aspect of liver disease management. The application of 2‐DG or the deletion of cell‐specific PKM2 can diminish the presence of specific CXCR3+ Th17 (ihTh17) cells in nonalcoholic fatty liver disease (NAFLD), ultimately mitigating the NAFLD driven by Th17 cells.^94^ Th22 cells secrete IL‐22, a cytokine primarily implicated in the onset and progression of autoimmune diseases, and it also facilitates the proliferation of hepatocellular carcinoma.^89^, ^95^

Treg, short for Tregs, is another subset of CD4^+^ T cells marked by the presence of the transcription factor forkhead box P3 (Foxp3).^96^ Tregs are activated and sustained through stimulation from factors like transforming growth factor (TGF)‐β, microbial‐associated molecular patterns, and metabolites, which are influenced by the liver‐brain‐gut neural axis.^97^ Tregs play a vital role in preserving immune tolerance by producing IL‐10, IL‐25, and TGF‐β to suppress T cell activation. They also produce cytotoxic T lymphocyte (CTL)‐associated antigen (CTLA)−4 and CD39 to regulate the behavior of B cells and DCs.^98^, ^99^ Due to Treg's immunomodulatory influence, it has garnered increased attention in studies concerning tumor microenvironments and immune tolerance. In a dual‐tumor immunocompetent mouse model, Lee and colleagues observed the activation of Tregs in conjunction with CD11b^+^ monocytes within the tumor, which contributes significantly to the diminished efficacy of antiprogrammed death‐ligand 1 (PD‐1) treatments.^100^ More frequently, Tregs collaborate with Th17 cells to modulate the immune response. In mice lacking Liver X receptor, there was a simultaneous increase in mesenteric lymph node (MLN) Th17 and RORγt+ Tregs, resulting in a more pronounced disease progression.^101^ The immune regulatory capabilities and plasticity of Tregs have elucidated their pivotal role in the onset and progression of malignant tumors, autoimmune diseases, and other pathological conditions.^102^ In upcoming years, there will be a progressive exploration of novel therapies directed at modulating Tregs for disease treatment. Notably, in conditions such as graft‐versus‐host disease, Tregs showcase compelling regulatory prowess, positioning them as a promising frontier in the realm of clinical immunotherapy.^103^, ^104^

CD8^+^ T lymphocytes, also referred to as CTLs, are primarily responsible for eliminating virus‐infected cells and contributing to inflammation.^105^ Typically, DCs and KCs in the liver can induce tolerance in CTLs by releasing IL‐10 and TGF‐β.^106^, ^107^ Furthermore, hepatic stellate cells and liver sinusoidal endothelial cells (LSECs) can induce immune tolerance in CTLs through the action of TGF‐β or cross‐antigens.^108^, ^109^ Compared with CD4^+^ T cells, CTLs are subject to greater control by immunomodulatory cells. Remarkably, chronic hepatitis B mouse models exhibit low levels of HBV‐specific CD8^+^ T cells, which leads to weakened immunity, largely due to the presence of arginase and indoleamine 2,3‐dioxygenase produced by immunomodulatory cells.^110^ In liver diseases, such as nonalcoholic steatohepatitis (NASH), IL‐15‐regulated CXCR6^+^ CD8^+^ T cells have been identified, and they accumulate in the liver where they target liver cells.^111^ To sum it up, T cells consist of diverse subsets that contribute to immune regulation and the execution of cellular immunity in the liver.

#### 2.2.2 B cells

B cells are involved in adaptive immune responses, primarily through humoral immunity in the human body, and they also contribute to immune regulation.^112^ B cells, which express CD19, are distributed within the liver as B1 cells, B2 cells, mature cells, immature cells, and plasma cells.^113^ When the B‐cell receptor (BCR) on the surface of thymus‐dependent B cells is triggered by an antigen, B cells and Th cells collaborate to generate antibodies through the interaction between CD40 and its ligand, facilitating humoral immunity.^114^, ^115^ A different category of thymus‐independent B cells carries out immune functions by attaching to antigens either through TLRs or by cross‐linking the BCR.^116^ Additionally, within the realm of B cell classification, there exists a subset known as regulatory B cells (Bregs) expressing CD19^+^ CD24^hi^CD38^hi^.^117^ Bregs serve as immunomodulators by releasing cytokines such as IL‐10, IL‐35, and TGF‐β.^118^, ^119^ An abundant presence of CD5^high^CD24^−^CD27^−/+^CD38^+/high^ TIM^−/+^ Bregs within tumor cells displaying the CD5^high^CD24 phenotype can elevate IL‐10 levels and suppress the killing activity of CD8^+^ T cells.^120^ B cells collaborate extensively with T cells in immune functions. Sequencing of B cell and TCRs revealed a substantial increase in the expression levels of T cell activation markers PD‐1 and CD38 in autoimmune hepatitis.^121^, ^122^ At the same time, single‐cell sequencing of NASH mouse livers revealed that B cell activation contributes to T cell‐associated inflammation by elevating the expression of the innate adaptor, myeloid differentiation primary response protein 88 (MyD88).^123^

Early after LT, two immunological processes take place: (1) donor liver resident cells enter the recipient's bloodstream, and (2) recipient immune cells infiltrate the transplanted liver.^124^, ^125^, ^126^ In a two‐phase scenario, LT is associated with AR and IRI.^127^ During this period, the activation of innate immune cells results in inflammatory damage, while the activation of adaptive immune cells leads to subsequent harm. In the later stages of transplantation, immune cells transition into immunosuppressive forms and participate in liver regeneration.^128^

## 3 IMMUNE EFFECTS IN LIVER IRI

Hepatic IRI is a recognized issue that impacts the success of LT.^129^ Factors like age and fatty liver can influence the extent of IRI and trigger AR.^129^, ^130^, ^131^ Hepatic injury linked to ischemia–reperfusion can be categorized into warm IRI and cold IRI.^132^, ^133^ IRI is connected to a decrease in ATP, disruptions in the mitochondrial respiratory chain, and the subsequent release of damage‐associated molecular patterns (DAMPs). These DAMPs can trigger KCs, neutrophils, and other innate immune cells, leading to an amplification of inflammatory responses.^134^, ^135^ In addition to inflammation and cell death, activated KCs and DCs also serve as APCs to interact with T and B cells, further worsening the damage^136^, ^137^ Additionally, immune cells play a role in guiding repair and fostering immune tolerance during the later stages of IRI.

### 3.1 Innate immune activation and response

#### 3.1.1 KCs activation and neutrophil extracellular traps

The generation of reactive oxygen species (ROS) and the activation of KCs depend on hypoxia‐induced mitochondrial dysfunction and ATP depletion.^138^ Mitochondria are key organelles in the respiratory transmission chain. In the context of IRI, mitochondria generate ROS as a result of disruptions in the electron transport chain, and this process is a major trigger for the activation of KCs.^139^ Following ROS activation, KCs persist in releasing ROS, exacerbating IRI. By producing extracellular ROS, the cytotoxicity of KCs in IRI cannot be ignored.^140^ Therefore, a key approach in treating IRI is the elimination of ROS to inhibit KCs activation. One of the most extensively studied pathways related to ROS reduction is the phosphatidylinositol‐kinase/protein kinase B (PI3K/AKT) signaling pathway. Activation of the PI3K/AKT pathway can enhance the production of heme‐oxygen‐1 (HO‐1) and alleviate the IRI process.^141^ The HO‐1‐associated gene is nuclear factor erythroid 2‐related factor 2 (Nrf2). Overexpressing tissue inhibitor metalloproteinase 3 (Timp3) or disrupting Rho associated coiled‐coil containing protein kinase 1 can successfully reverse the liver injury caused by Nrf2 knockout in mice.^142^ Melatonin, which is an oxygen free radical scavenger, has also been shown to reduce ROS production and protect mitochondrial function.^143^ Furthermore, silencing methylation‐controlled J protein can enhance mitochondrial activity, decrease ROS production, and trigger KCs‐mediated liver regeneration.^144^

The primary inflammatory responses of KCs are elicited through the pattern recognition receptors (PRRs) on KCs, as well as the danger‐associated molecular patterns released by deceased hepatocytes, which mediate the innate immune response.^145^, ^146^ Hepatocytes undergo pyroptosis during hepatic IRI. Research has revealed an upregulation of nucleoside binding oligomerization domain 1 (NOD1) expression in AML12 mouse hepatocytes exposed to IRI. The up‐regulation of NOD1 expression is accompanied by Caspase‐1, gasdermin D, and IL‐1β and other markers of pyroptosis and inflammatory factors were up‐regulated.^147^ This study implies a therapeutic avenue: inhibiting genes associated with pyroptosis to mitigate the progression of IRI. PRRs include four classes, among which TLRs have been most well studied in macrophage‐mediated IRI.^148^, ^149^ The receptors that engage with PRRs to trigger KCs are primarily endogenous damage‐associated and/or pathogen‐associated molecular patterns (DAMPs/PAMPs) generated predominantly by apoptotic hepatocytes.^150^, ^151^ The two factors interact to guide downstream reactions. Among the IRI signaling pathways involved in KCs, the high mobility group box‐1 (HMGB1)/TLR4 axis is the most classic. HMGB‐1 has the best specificity for DAMPs released during hepatic IR and interacts with TLRs, especially TLR4, to activates it.^152^, ^153^ Additional DAMPs encompass DNA, ATP, urate, mitochondrial formyl peptides, and S100 proteins.^154^ TLR4 is activated and mediates MyD88‐dependent and MyD88‐independent pathways.^155^, ^156^ Several downstream factors, driven by these two pathways, including nuclear factor kappa‐B (NF‐κB), activator protein‐1, IFN regulatory factor 3, signal transducer and activator of transcription 1 (STAT‐1), and other proinflammatory factors, contribute to the formation of the inflammatory microenvironment.^157^, ^158^, ^159^, ^160^ Targeting alternative signaling pathways also has a positive effect on alleviating IRI. Serelaxin can be targeted to increase KCs and Notch expression, thereby reducing neutrophil and macrophage infiltration.^161^, ^162^ Medications aimed at the HMGB1/TLR‐4 pathway have been created for the management of IRI, including drugs like aucubin and salicylate acetyl‐3‐aminoethyl salicylic acid (ac3AESA).^163^, ^164^ Moreover, pathways associated with the receptor for advanced glycation end products (RAGE) are also implicated in the generation of chemokines (CXCL)2 and other inflammatory cytokines.^165^ Activated KCs function as immune cells driving predominant inflammation in IRI, releasing abundant ROS and proinflammatory cytokines like TNF‐α, IL‐1, INF‐γ, and IL‐12.^166^, ^167^ Furthermore, the complement system components C3a and C5a are elevated in IRI, providing an additional stimulus for the release of ROS, TNF‐α, and IL‐1 by KCs.^168^ In late stage, KCs mediates hepatocyte regeneration by stimulating regenerative signaling pathways and secreting inflammatory and growth factors. KCs can promote hepatocyte repair through Wnt derivation during liver injury.^169^, ^170^ Additionally, in the early stages of injury, KCs function as inflammatory cells, releasing TNF‐α and IL‐6, which contribute to inflammatory damage and, in turn, activate the regeneration program.^171^ TNF‐α and IL‐6 are regarded as key cytokines for regeneration. These factors activate STAT‐3 and promote cell proliferation at the gene level.^172^ Moreover, TGF‐β1 expressed by KCs affects liver regeneration through the TGF‐β signaling pathway.^173^ Bird et al.^174^ discovered that inhibiting TGF‐βR using a TGFβ receptor 1 (TGF‐βR1) inhibitor effectively mitigated aging and enhanced liver regeneration. These findings underscore the significant potential of KCs in promoting liver regeneration.

DAMPs also activate neutrophils, and KCs, which are initially activated, further enhance neutrophil activation. Neutrophils are influenced by TNF‐α and IL‐1 produced by activated KCs, leading to an upregulation of neutrophil adhesion protein MAC‐1 (CD11b/CD18), inducing IL‐8 synthesis and further enhancing neutrophil chemotaxis.^175^ In response to IL‐1 stimulation, the inflammatory factor TNF‐α can upregulate intracellular adhesion molecule‐1 and p‐selectin expression within the LSEC, subsequently promoting the recruitment of rolling neutrophils and the production of ROS.^176^, ^177^ Inflammatory cytokines such as CXCL1, CXCL2, and CXCL5 play a significant role in neutrophil‐mediated responses during IRI. Neutralizing these cytokines effectively reduces neutrophil chemotaxis and inhibits the development of IRI.^178^ Activated neutrophils generate neutrophil extracellular traps (NETs) and participate in pathways associated with DAMPs via TLR4 or TLR9.^179^ The release of IL‐3 from hepatic sinuses exacerbates the production of NETs, subsequently promoting the transition of macrophages to a proinflammatory phenotype.^180^, ^181^ Research findings confirm this association, as the knockdown of human antigen R substantially reduces the expression of surface markers on neutrophils and macrophages, and it exerts a protective effect against HO‐1 in the liver.^182^ Hepatocellular cyclooxygenase‐2 (COX2) and recombinant human liver regeneration enhancer can significantly diminish the levels of inflammatory mediators and disrupt TLR4 signaling, leading to the inhibition of neutrophil infiltration and macrophage‐driven inflammation.^183^, ^184^ Significantly, Zucoloto et al. reported that NET formation promotes platelet aggregation, contributing to thrombus formation, with involvement of DNA, histones H3, and H4.^185^

#### 3.1.2 Dendritic cells

Similar to KCs, immature DCs are first activated by DAMP and PAMP following LT.^186^ DCs become activated in IRI when their surface PRRs bind to DAMPs and PAMPs. This activation leads to the upregulation of MHC, CD80, and CD86 expression, as well as the production of inflammatory cytokines such as TNF‐α and IL‐12.^187^, ^188^ The content of pDCs was notably reduced in mice lacking the Flt3 ligand (Flt3L), and this reduction was associated with decreased liver damage.These findings suggest that Flt3L might be a potential target gene for the treatment of IRI.^189^ Li et al. also discovered that the surface protein T‐cell immunoglobulin‐domain and mucin‐domain 4 (TIM‐4) plays a role in regulating DCs. Blocking TIM‐4 can enhance the production of CD4^+^ CD25^+^ Foxp3^+^ Tregs and mitigate IRI.^190^ Nevertheless, DCs in IRI play a dual role, not only in provoking a proinflammatory response but also in promoting immune tolerance. Loi et al. observed that hepatic DCs matured further following IRI, leading to the production of IL‐10, an anti‐inflammatory cytokine that reduces the cytotoxic effects of NK cells and T cells.^191^ Bamboat et al. observed an excessive depletion of cDCs in the early stages of mouse IRI. They found that transplanting cDCs into the liver activated TLR9 and led to the production of IL‐10.^192^ This suggests that DCs could be a potential target for mitigating inflammation in IRI and fostering immune tolerance. Additional investigations also validated the involvement of immune‐regulating proteins associated with DCs. Analysis through polymerase chain reaction and flow cytometry revealed that DAP12^−/−^ mice exhibited increased IRI, along with heightened activation of hepatic DCs. Interestingly, infusing wild‐type mice with DCs did not ameliorate the condition.^193^ The prostaglandin E receptor (EP)3, expressed on the surface of DCs, has been discovered to produce the anti‐inflammatory cytokine IL‐13. This, in turn, inhibits the shift of macrophages toward inflammatory phenotypes.^194^ DCs display a complex dual role in IRI. In the context of cellular immunotherapy, a novel approach involves targeting IRI posttransplantation using DCreg.^195^ DCregs are generated from immature DCs that phagocytose apoptotic DCs and inflammatory factors, including IL‐10.^196^, ^197^ Unlike other DCs, DCregs exhibit lower levels of CD80 and CD86 expression but possess higher levels of PD‐L1. They also release IFN‐γ, IL‐17, and IL‐10, which only weakly stimulate CD4^+^ and CD8^+^ T cells.^198^ Conversely, DCregs can promote the generation of Tregs, offering a new approach to sustaining tolerance in LT. However, administering allogeneic DCregs to organ transplant recipients may pose lethal risks.^199^ Clinical studies by Camila et al. revealed that infusing DCregs into living donor LT (LDLT) patients led to elevated levels of Foxp3^+^Tregs and T cell suppression.^200^ Consequently, ensuring the effectiveness of DCregs infusion remains a significant challenge in clinical practice.

#### 3.1.3 NK and other innate immune cell

An increasing number of reports have substantiated the involvement of NK cells in IRI. In the early stages of rat LT, there was a notable rise in the expression of NKG2D on the surface of NK cells, along with the production of proinflammatory cytokines TNF‐α, IL‐1β, and IL‐6, as well as cytotoxic molecules perforin and granzyme B.^201^ NK cell activation results in the production of IFN‐γ, which can cause harm by increasing Fas expression in hepatocytes and synergizing with IL‐18 released by KCs.^149^, ^202^ Besides, IL‐17 is another factor influencing NK cell production. Depletion of NK cells in Rag1^−/−^ mice leads to a decrease in IL‐17 levels, along with a reduction in neutrophil chemotaxis.^203^ Nonetheless, NK cells continue to have an immunoregulatory role. In a study comparing the phenotype of NK cells in the recipient liver, Jamil et al.^204^ observed that the absence of STAT4 phosphorylation results in the development of tolerant NK cells, thereby mitigating damage after transplantation. KIRs have been investigated for their role in binding to HLA and modulating the immune functions of NK cells.^205^ IL‐10 can decrease the production of chemokines CXCL9, CXCL10, and CXCL11 by DCs, leading to a reduction in NK cell infiltration and the suppression of their cytotoxic effects.^206^ There are reports indicating that liver NK cells derived from donors exhibit immunotolerance.^207^ there is ample potential for the advancement of immunotherapies involving NK cells. Additionally, other innate immune cells, including NKT cells, especially the Valpha14 NKT cell subtype, have been recognized for their role in immune tolerance following infusion.^208^ In IRI, platelets are influenced by the inflammatory mediators released by KCs, resulting in the release of IL‐6, platelet‐activating factors, growth factors, TGF‐β, and other cytokines that contribute to the advancement of inflammation.^209^, ^210^, ^211^ Peritoneal macrophages with the F4/80^hi^GATA6^+^LMP phenotype have been observed to migrate to the injury site and contribute to liver repair in experimental models of acute liver injury.^212^

### 3.2 Adaptive immune response led by T cells

T cells are activated by innate immune cells during the later stages of liver IRI and play a significant role in the ensuing phase. CD4^+^ T cells are involved in the reciprocal activation of KCs and present potential targets for mitigating inflammation and promoting liver repair in the later stages.^213^ In IRI, CD4^+^ T cells are activated by IL‐6 and TNF‐α from KCs, which further activate KCs.^214^ When exposed to TNF‐β, macrophage colony‐stimulating factor, and IFN‐γ generated by CD4^+^ T cells, KCs undergo further activation, leading to the initiation of neutrophil‐mediated inflammation.^215^, ^216^ It has been demonstrated that CD4^+^ T cells are biactively activated through the costimulatory receptor CD154–CD40 interaction with APCs, and blocking CD154 can mitigate the damage caused by CD4^+^ T cells.^213^, ^217^ Nrf2 is also present in CD4^+^ T cells and hinders their transformation into proinflammatory phenotypes such as Th1 and Th17. This, in turn, helps prevent inflammatory damage mediated by CD4^+^ T cells by regulating CD4^+^ T cell‐related genes. The use of normothermic machine perfusion (NMP) can additionally reduce the production of inflammatory CD4^+^ T cells at the source, making it an effective clinical approach.^218^ It's important to note that CD4^+^ T cells are influenced by innate immune cells and later transition into Treg forms. In T cell responses involving KCs, the production of Tregs is also regulated by KCs, contributing to the inhibition of inflammation.^219^, ^220^ Neutrophils, among other innate immune cells, can also contribute to the environment that supports Tregs production by generating ROS.^221^ When CD4^+^ T cells and platelets were cocultured in vitro, a reduction in T cell‐derived IFN‐γ, TNF‐α, and IL‐6, along with an increase in Foxp3^+^ T cells, were observed. This suggests that platelets may also have a regulatory role in the IRI environment.^222^ At present, the inflammatory role of adaptive immune cells in LT‐related IRI is rarely reported, the focus has primarily been on rejection. However, there is a growing interest in their late‐stage immunomodulatory roles.

**FIGURE 3:**
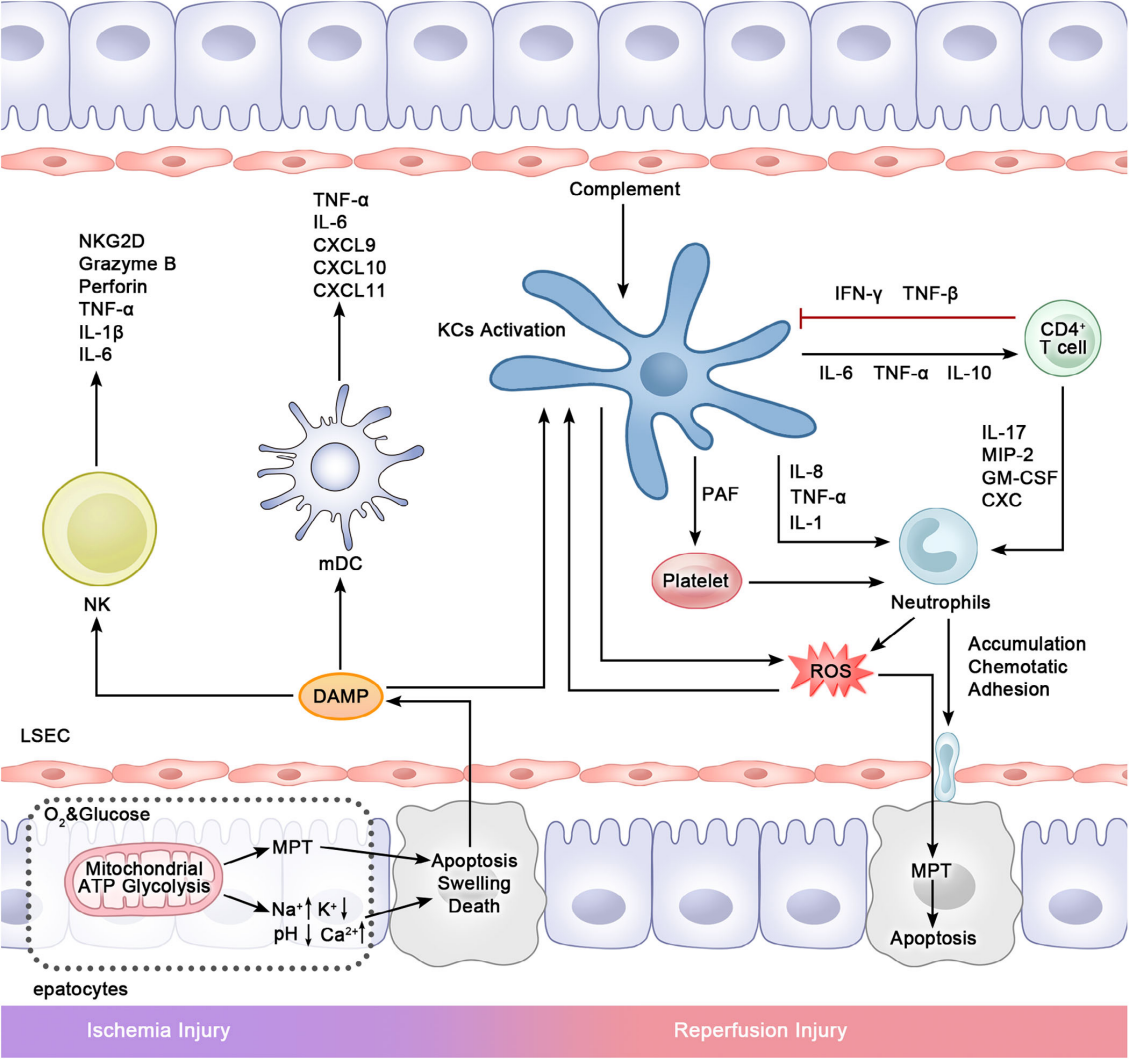
Hepatic immune microenvironment at IRI stage. Hypoxia leads to mitochondrial dysfunction and apoptosis of hepatocytes. Factors released by dead hepatocytes, mainly PAMPs, induce activation of KCs, mDCs, neutrophils, and NK cells. Activated immune cells release inflammatory cytokines involved in injury, causing cell damage. KCs releases ROS and stimulates both neutrophils and CD4^+^T cells, resulting in subsequent damage. Platelets are also affected by KCs to promote neutrophil aggregation. On the other side, CD4^+^T cells release inflammatory cytokines that inhibit KCs activation. PAF, platelet activating factor; CXC, chemokine receptors.

In summary, IRI represents the initial phase following LT. During this process, innate immune cells, notably KCs and neutrophils, serve as the primary early effectors of tissue damage and become activated through the release of ROS and DAMPs/PAMPs. Additionally, other innate immune cells like DCs are also prompted into proinflammatory states by DAMP activation. In the presence of this inflammatory environment shaped by innate immune cells, T cells, the primary late‐stage effectors, are influenced to promote inflammation and cell destruction. Consequently, reducing the generation of ROS and DAMP during perfusion could be a promising strategy for future treatments. This approach could effectively mitigate the inflammatory response associated with liver IRI and prevent subsequent immune rejection (Figure 3).

## 4 IMMUNE RESPONSE TO AR

Rejection following LT can be categorized into AR and chronic rejection (CR). Unlike other vascularized organ transplantations, the occurrence of CR after LT is relatively low, at just 3−5%.^223^ So, in this section, we will concentrate on AR. AR is a significant complication that impacts the outcome of LT and develops in about 30% of patients within the first 12 months posttransplantation.^224^ By combining the Banff schema with CT imaging and blood markers, a definitive diagnosis of AR can be established.^225^, ^226^, ^227^, ^228^ In AR after LT, immune cells are involved in two primary processes: (1) inflammation resulting from preservation–reperfusion injury (PRI), and (2) APCs triggering adaptive immune responses based on the MHC, which then involves (3) adaptive immunity with T cells as the primary effectors.^229^, ^230^, ^231^

### 4.1 Preservation–reperfusion injury

PRI is essentially the consequence of graft ischemia due to alterations in blood supply during cooling, now often termed as cold IRI.^232^ PRI encompasses ROS production, ATP depletion, mitochondrial dysfunction, calcium overload, and subsequent hepatocyte impairment.^233^ Following this, innate cells become the primary activators, resulting in subsequent inflammatory damage.

Innate immune cells, particularly KCs and neutrophils, can be primarily activated by dead hepatocytes through the production of DAMPs.^234^ Activated KCs initiate the initial impact on the donor liver by releasing inflammatory cytokines, creating an immune microenvironment that triggers a lymphocytic response.^235^, ^236^ In response to inflammatory factors, KCs, LSECs and CD4^+^ T cells form an interconnected inflammatory response network.^237^, ^238^ PRI also leads to an increase in MHC expression on APCs, such as KCs and DCs, which subsequently triggers additional responses.^239^

Therefore, another effective approach to enhance the prognosis of LT involves focusing on the preservation of the donor liver and minimizing the activation of immune cells that cause inflammation. In recent years, there has been increased attention to optimizing cryopreservation techniques for donor livers. In a study exploring the pretreatment of solid organs with an adjunct hydrogen sulfide (H_2_S) preservation solution, Dugbartey et al.^240^ suggested that H_2_S could reduce ROS production associated with IRI, thereby preserving the activation of macrophages and other immune cells. This discovery introduces novel pharmacological considerations for the constituents of preservation solutions. Additionally, irrigating with heavy water (D_2_O) has been demonstrated to lower oxidative stress and suppress immune cell activation.^241^ Furthermore, enhancing preservation methods is a valuable strategy to explore. Hypothermic oxygenated perfusion, considered an advanced organ preservation technique, has been found to mitigate the NF‐κB inflammatory pathway downstream of HECTD3/TRAF3, consequently reducing IRI.^242^, ^243^ Tamaki et al. suggested using hydrogen irrigation following refrigeration as a novel in vitro treatment for end‐stage ischemia, aimed at mitigating oxidative damage resulting from ROS generation.^244^ Given the donor liver's condition, special attention is still required for certain donor liver preservation and reperfusion techniques. Modifying the ratio of polyethylene glycol 35 to glutathione in the preservation solution can effectively decrease the generation of mitochondrial oxidative phosphorylation complexes, mitochondrial uncoupling protein 2, and inflammatory ROS during cold preservation reperfusion.^245^ In conclusion, enhanced preservation and perfusion techniques can lower the generation of proinflammatory factors by immune cells, thereby suppressing cellular inflammation and ameliorating liver injury caused by PRI.

### 4.2 Innate immune cells: APCs and MHC‐related rejection

The disparity in MHC between the donor and recipient has long been a focal point in organ transplantation. In the early 1980s, Markus established a connection between HLA and rejection by studying over 500 liver transplant cases.^246^ These findings suggested a potential “dualistic” role of HLA in LT. In the healthy liver, cells with nuclei express MHC I, whereas MHC II is solely expressed in APCs. However, in response to PRI, both MHC I and II expression on cell surfaces are upregulated within the liver's inflammatory environment, promoting allograft rejection.^43^ Remarkably, due to its functional complexity, HLA has been demonstrated to also influence clinical immunosuppressive therapy and tolerance.^247^ HLA evolutionary divergence (HED) is utilized as a continuous measure to quantify peptide disparities between two related HLA alleles. Cyrille proposed that, by assessing the HED of HLA I and II in patients with AR, donor HLA I could serve as an indicator of AR as well as a guide for immunosuppressive therapy.^248^ Therefore, HLA assessment can provide an additional method for evaluating immunosuppression. HLA II has also been linked to later‐stage CR and can serve as a marker for late rejection.^249^, ^250^ MHC I has been known to promote tolerance in other organ transplantations, suggesting the potential for future gene therapy in liver transplant‐related AR.^251^ Additionally, MHC antibody therapy represents a significant clinical approach for managing AR.^252^ The main APCs in the liver, can capture foreign antigens and present MHC molecules through cytoplasm or exosomes to either activate or inhibit T cells.^13^, ^253^, ^254^, ^255^ Under the influence of proinflammatory factors like PRI, APCs in the donor liver upregulate their MHC expression, triggering T‐cell‐mediated immune responses.^106^, ^256^, ^257^ Early rejection responses are primarily driven by T cells and are characterized by cellular immunity. This is why acute allograft rejection is often referred to as T‐cell mediated rejection (TCMR).^258^ CD8^+^ T cells and CD4^+^ T cells, acting as effector cells responsible for TCMR, can be activated directly through the MHC pathway of graft APCs. Host APCs also indirectly activate CD4^+^ T cells by presenting antigenic peptides.^259^ The activation of T cells by APCs also encompasses a semi‐indirect pathway. This pathway involves T cell activation signaling, costimulatory receptors on T cells (CD28, CD40), corresponding ligands on APCs (CD40L, CD80, CD86), and the microenvironment that triggers T cell activation.^260^ KCs also mediates the T cell response and secondary apoptosis of other immune cells through cytokines.^261^, ^262^ Rather than always resulting in rejection, these APCs switch to assuming an immune tolerance role later in transplantation. Previous studies have shown that, in the livers of patients with advanced liver transplants, more KCs are replaced by the recipient's KCs, indicating that the recipient's own KCs possess the capacity to sustain immune tolerance.^263^, ^264^ Xu et al.^265^ discovered that the expression of scavenger receptor class F (SCARF)1 in KCs decreased during AR, and increasing SCARF1 expression could enhance the PI3K–AKT–STAT3 signaling pathway, consequently mitigating the AR process. Zhang et al. conducted a study revealing that the downregulation of X‐box binding protein 1 in KCs diminishes liver damage by inducing the transformation of macrophages into the M2 phenotype through Janus kinase 3 signaling.^266^ Unlike KCs, DCs play a crucial role in mediating rejection and are a significant target for immune tolerance therapy. In mouse models, it was observed that post‐LT, liver DCs were gradually replaced by host DCs. This shift was associated with elevated expression of PD‐L1 and IL‐10, both of which inhibited T cell activation.^267^ DAP12 (DNAX‐activating protein of 12 kDa) was discovered to reduce the expression of DCs in mouse liver allotransplantation. This reduction was accompanied by increased levels of TNF‐α, IL‐6, and other proinflammatory factors, ultimately enhancing T cell immunity.^268^ In different studies, the influence of DCs on donor receptors has been reversed. Transferring liver cDCs to an allogeneic pancreas can extend the duration of tolerance, indicating that donor liver DCs also possess anti‐rejection properties.^269^ However, DCregs, as a regulator of DCs' immune behavior, represent a novel clinical therapy for AR. Animal models have demonstrated the potential of DCregs to enhance organ transplant outcomes.^270^, ^271^ Clinical trials have demonstrated that infusing DCregs into liver transplant patients leads to elevated levels of donor immunomodulatory markers PD‐L1, CD39, and CD73. This is accompanied by an increase in CD25^hi^CD127^−^Foxp3^+^Tregs.^200^ DCregs, generated through a culture of vitamin D3 and IL‐10 followed by CD14 immune bead purification, exhibit reduced levels of CD80 and CD86 costimulation. They can effectively suppress CD8^+^ T cell responses, thereby improving the prognosis of LT.^198^ In the later stages of transplantation, CD4^+^ T cells home specifically to areas of AR or are released into the peripheral blood, becoming the primary drivers of immune tolerance.^272^

In summary, innate immune cells play a crucial role in antigen presentation by APCs in immune rejection associated with transplantation. Other innate immune cells, like NK cells, were also found to possess heightened cytotoxicity in transplanted donor CD56^bright^NK cells, which helps reduce rejection stemming from recipient T cell infiltration.^126^, ^273^ Innate immune cells maintain tolerance by considering cytokine action alongside antigen presentation and immune response. Inflammatory mediators, such as IL‐34, can induce immune cells to produce secondary immune tolerance.^274^ Tolerance cell therapy, such as DCreg, has been applied to achieve immune tolerance in AR through the cultivation of tolerance factors. Additionally, complement factor C4d has shown promise as a predictor of AR, highlighting the potential of the complement system in LT regeneration.^275^ This also represents a novel avenue for future immunosuppression and treatment.^276^

### 4.3 Adaptive immune cells: TCMR and regulation

Activated by APCs, CD4^+^T cells can differentiate into a variety of phenotypes, including Th1, Th2, Th17, and Tregs. In AR, Th1 and Th17 participate in inflammation and secrete TNF‐α, IFN‐γ, and IL‐2 to promote rejection.^277^, ^278^ Due to positive feedback, the population of Th1 cells can further expand. Activated by MHC I, CD8^+^ T cells serve dual roles: they transform into cytotoxic T‐cells (Tc) to induce cell death via the perforin/granase pathway and Fas–FasL pathway, while also promoting the proliferation of Th1 cells.^279^ Th2 cells, opposing Th1, can produce IL‐4 and IL‐10 to suppress the development of immune rejection.^280^, ^281^ Therefore, the Th1/Th2 ratio can influence the course of AR to some degree. Additionally, in current research, the Tregs/Th17 ratio is considered an effective predictor of LT outcomes.^282^, ^283^ Previous studies demonstrated that administering Tregs to mice with acute liver injury can ameliorate inflammation and elevate IL‐10 levels.^284^ Furthermore, CD4(+)CD25(+)FoxP3(+)Tregs were observed to accumulate in immunologically tolerant rats following total lymphoid irradiation.^285^ Peripheral blood Tregs in LT patients showed a higher presence of CD45^RO+^Tregs (activated Tregs, aTregs), indicative of immune regulation activation.^286^ All of the above findings confirm that Tregs can mitigate inflammation and enhance immune responses in both animals and humans. While a majority of Tregs originate from the thymus, some are generated through differentiation of CD4+T cells induced by MHC I or TGF‐β during LT.^251^, ^287^ Tregs, as immunoregulatory cells, suppress T cell activity by releasing inhibitory cytokines like IL‐10, IL‐35, and TGF‐β, and through the perforin/granase pathway. Additionally, Tregs utilize ATP via CD39/CD73 and employ CTLA‐4 to facilitate CD80/86 internalization on APCs, thereby competing with T cells for IL‐2 and preserving immune tolerance.^288^, ^289^ As a result, Tregs have emerged as a novel approach for promoting tolerance after organ transplantation. They have demonstrated effective immune tolerance and safety in other organ transplantations, including kidney transplantation.^290^ In essence, Treg cells were isolated from liver transplant recipients using the Good Manufacturing Practice technique, sorted with CliniMACS, and then cultured with IL‐2 and rapamycin for amplification.^291^, ^292^ Alberto and his team demonstrated the safety of Treg cells by infusing them at doses of 50,000–1 million Tregs per kilogram, or 3–4.5 million Tregs per kilogram, in six patients who consented to treatment 6–12 months after transplantation.^293^ In recent years, Safinia and colleagues conducted a combined Phase I/IIa clinical trial, ThRIL, for Treg immunotherapy after LT (NCT02166177). Additionally, a clinical trial employing Tregs to induce liver transplant tolerance in both living (NCT02474199) and deceased donors (NCT02188719) is being carried out at the University of California, San Francisco, USA. These clinical studies collectively demonstrate the feasibility of using Tregs in clinical LT tolerance (Table 1).

**TABLE 1:** Clinical study of immune cell infusion for liver transplantation in recent 10 years.

Study ID	Phase	Treatment	Country	Biological origin	Transplantation indication	Dose	Status
NCT04208919	I/II	DCregs	USA	Donor‐derived DCreg	LDLT	3.5–10 × 10^6^/kg	Active, not recruiting
NCT03164265	I/II	DCregs	USA	Donor‐derived DCregs	LDLT	Unclear	Active, not recruiting
NCT03654040	I/II	Tregs	USA	Single dose of Treg product (arTreg)	LT	90 × 10^6^	Terminated
NCT03577431	I/II	Tregs	USA	Single dose of Treg product (arTreg‐CSB)	LT	2.5–125 × 10^6^	Active, not recruiting
NCT02474199	I	Tregs	USA	Donor Alloantigen Reactive Tregs(darTregs)	LT	300–500 × 10^6^	Completed
NCT02188719	I	Tregs	USA	Donor Alloantigen Reactive Tregs(darTregs)	LT	25–60 × 10^6^, 100−240 × 10^6^, 400−960 × 10^6^	Terminated
NCT01624077	I	Tregs	France	Donor‐derived DCregs	LT	Unclear	Not yet recruiting
NCT01624077	I	Tregs	China	Donor‐derived DCregs	LT	1 × 10^6^/kg	Unknown status

*Data sources*: www.clinicaltrials.gov.

**FIGURE 4:**
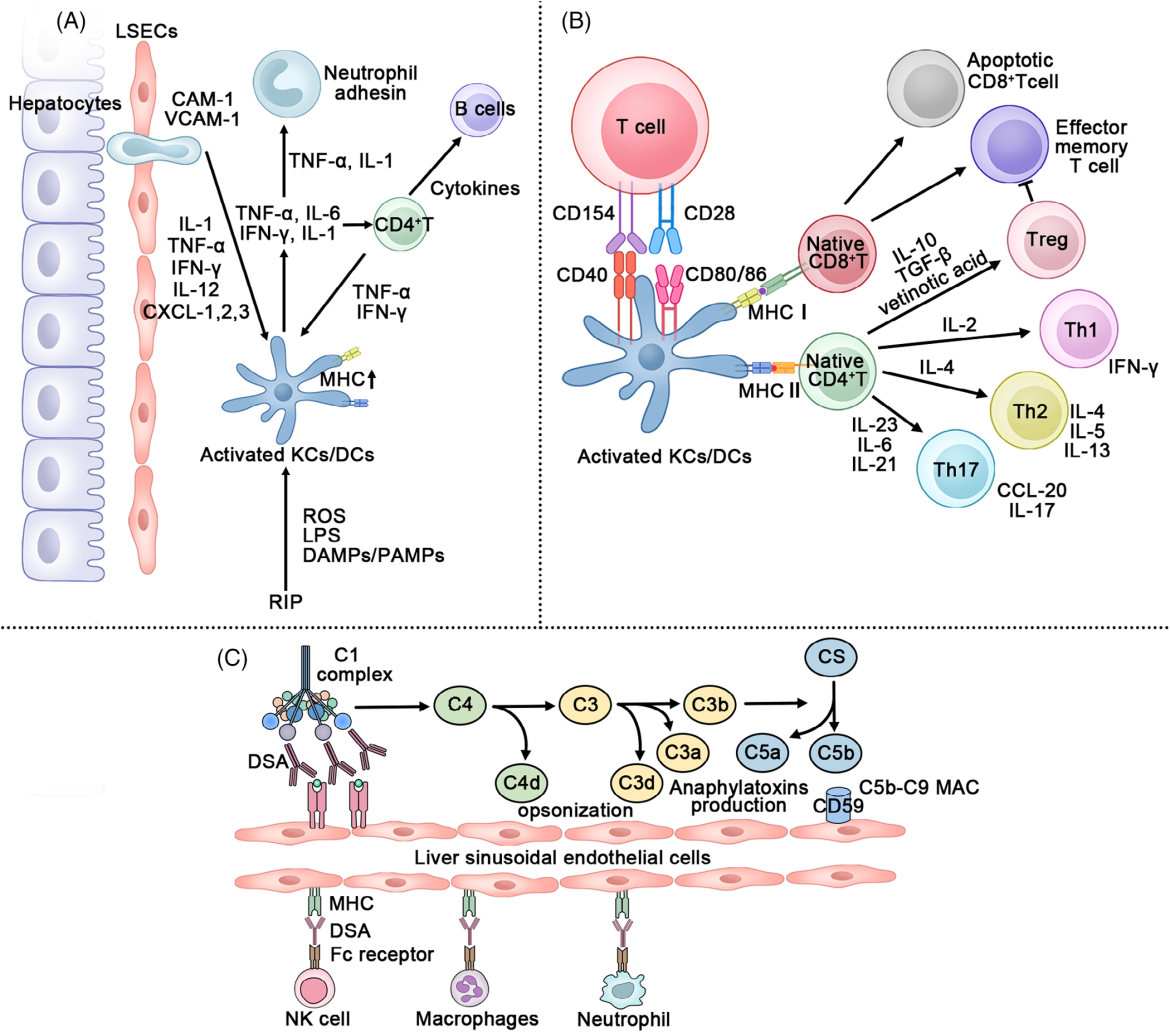
Three pathways of immune rejection after liver transplantation:(A) Preservation‐reperfusion injury (PRI)‐based acute rejection: PRI is an inevitable stage in liver transplantation. ROS, DAMPs, PAMPs, and lipopolysaccharide (LPS) produced during PRI can activate KCs to release inflammatory mediators and upregulate the expression of MHC I and II. PRI therefore facilitates AR. (B) MHC‐based acute rejection: AR occurs due to MHC I and II on activated donor KCs. On the one hand, the MHCI on KCs and the costimulatory receptors CD40 and CD80/86 are involved in the activation of CD8^+^ T cells. Participation in TCMR occurs. On the other hand, KCs are involved in CD4^+^ T‐cell activation through MHC II antigen presentation, which is related to late immune tolerance. (C) Antibody‐mediated rejection: After the DSAs bind to MHC antigens on the liver allogeneic, the classical pathway of complement activation is through the binding of C1 complex. Cell damage is caused by subsequent complement reactions. In addition, DSA can bind MHC molecules and promote the aggregation and damage of innate cells such as neutrophils, macrophages, and NK cells through association with FC receptors. CAM, cell adhesion molecule. VCAM, vascular cell adhesion molecule; C, complement.

In clinical practice, the incidence of liver AR remains relatively high. During the PRI phase, innate immune cells such as KCs and DCs act as APCs, causing inflammatory damage while increasing the expression of surface MHC molecules. Different types of T cells are subsequently activated through MHC antigen presentation, leading to their role in cell killing and inflammation. In addition to these processes, one cannot ignore a rare category of antibody‐mediated rejection (AMR) driven by donor‐specific antibodies (DSAs). Currently, two major hypothesized mechanisms of AMR involve DSA activation through the complement classical pathway and injury mediated by Fc receptors.^239^, ^294^ Currently, clinical strategies for reducing postoperative AR include improvements in perfusion instruments and perfusion fluids, the use of immune cell inhibitors, and the delivery of regulated immune cells. Nevertheless, there is ample room for further exploration of these treatment approaches (Figure 4). Furthermore, immune imbalance following LT may lead to associated complications such as gut dysbiosis, AKI, biliary tract issues, and tumor recurrence.

## 5 LT AND GUT DYSBIOSIS

Infection after LT is a serious complication. Infection can occur up to 80% of the time after LT.^295^, ^296^ In the context of posttransplantation infections, beyond the inherent risk of hepatitis virus recurrence in transplant recipients, a prominent factor contributing to infection susceptibility is the presence of intestinal bacterial infections.^297^, ^298^ In recent years, particularly in individuals with cirrhosis, there has been a progressive annual rise in the occurrence of invasive fungal infections.^299^ This has also motivated us to intensify our focus on postoperative drug utilization and preventive management in clinical settings. Given that dysbiosis and translocation of intestinal bacteria stand as primary contributors to infections post‐LT, this section predominantly delves into the infections arising from intestinal flora imbalance and outlines the advancements in their treatment.

### 5.1 Infection caused by gut dysbiosis

Infection remains a significant factor contributing to increased mortality after LT. The primary culprits behind postoperative infections are an imbalanced intestinal microbiome and colonization by multidrug‐resistant bacteria, often triggered by drug use, particularly antibiotics.^300^ Furthermore, the intestinal barrier function in liver transplant recipients is more susceptible to damage, potentially resulting in bacterial translocation or overgrowth. This vulnerability can be attributed to various factors, including preoperative end‐stage liver disease and the intraoperative absence of hepatic blood flow blockage.^300^, ^301^ The gut hosts a diverse microbiome, encompassing bacteria, fungi, and viruses. This microbiome exerts its influence by producing various secondary metabolites through the breakdown of intestinal contents.^302^ Among these metabolites, short‐chain fatty acids, various branched‐chain amino acids, and secondary bile acids (BAs) produced through cellulose fermentation offer some of the most significant benefits.^303^, ^304^, ^305^ Butyrate, a byproduct of dietary fiber fermentation by intestinal microbes, has been shown to enhance intestinal integrity and mitigate liver disease.^306^ Furthermore, the intestinal microecology plays a role in preserving immune equilibrium by triggering innate immune receptors like TLRs and IgA.^307^, ^308^ In response to the microbiota's influence, these metabolites traverse the intestinal epithelium, facilitated by immune factors released by the intestinal immune system. They then enter the circulatory system, where they exert their functional effects on various host tissues and organs, contributing to the creation of a unique immune microenvironment.^309^ However, detrimental bacteria within the gut microbiota can jeopardize host health by producing LPS, bacterial toxins, and metabolites.^310^ This regulatory mechanism governing the interplay between gut flora and host organs is commonly referred to as the “gut axis.” There exists a close connection between the liver and this “gut axis,” giving rise to the concept of the liver–gut axis, which holds significant implications for LT and disease progression.^311^, ^312^

The gut microbiota's impact on liver disease involves interactions with the innate immune system. Gut microbiota antigens or immunomodulatory metabolites are used to stimulate and activate DCs to counteract the immune tolerance induced by immature DCs.^313^ Administering Bifidobacterium orally heightened DCs activation, consequently boosting CD8^+^ T cell responses. Additionally, DC and IL‐12‐dependent Th1 cells stimulated by Bacteroides flimsii can enhance the blocking of CTLA‐4.^314^, ^315^ In innate immune cells, KCs are closely associated with infections resulting from intestinal dysbiosis. Under typical circumstances, KCs efficiently eliminate microbially derived molecules, like LPS, from the intestine that enter the liver through the bloodstream.^316^ Metabolites generated by the gut microbiota, including high‐density lipoprotein and tryptophan, influence the activation of hepatic macrophages, consequently impacting the development of liver diseases.^317^, ^318^ These factors are intricately linked via the gut–liver axis, playing a role in sustaining normal liver function and influencing the course of diseases. After LT, liver damage and the use of immunosuppressive drugs weaken the regulation of intestinal flora and the ability of liver macrophages to fend off harmful bacteria. This leads to the activation of liver macrophages into the proinflammatory M1 type, which becomes prominent during infections.^295^, ^319^ Hence, the development of imbalances in intestinal microbiota and liver inflammation posttransplantation should not be underestimated. Nevertheless, conversely, gut microbiota can enhance macrophage characteristics following transplantation. Research has demonstrated that intestinal microorganisms secrete cathepsin K, which facilitates M2 macrophage polarization through the TLR4 pathway and regulates immune responses.^320^ The intestinal flora also plays a crucial role in BA conversion, converting primary BAs into secondary BAs.^321^ The Farnesoid X receptor (FXR) and Takeda G‐protein coupled receptor‐5, both widely expressed on monocytes and macrophages, control inflammation and the storage of BAs by interacting with secondary BAs.^322^ Following a liver transplant, there's a surge in primary BAs accumulation that disturbs the balance of BAs, leading to the activation of macrophages and triggering inflammation. However, modifying the microbiota and boosting the levels of secondary BAs can reverse this effect.^323^, ^324^ Gene sequencing and metabolomics have also unveiled that various other metabolites produced by intestinal microbes, like 3,4‐dihydroxyphenylpropionic acid (3,4‐DHPPA), have the potential to suppress macrophage activation and alleviate IRI in mice.^325^ In conclusion, it's also essential to consider the examination of the intestinal flora and macrophages. The exploration of beneficial strains for potential clinical applications can be a promising avenue for future research.^326^, ^327^, ^328^

Tregs are pivotal in establishing and preserving immunological tolerance to both self and alloantigens, thereby enhancing the overall tolerogenicity of liver transplants. The mesenteric lymph nodes, serving as the crucial connection between intestinal symbiotic bacteria, constitute a vital component of intestinal immunity.^329^, ^330^ Consequently, dysbiosis resulting from LT, including alterations in the composition of intestinal symbiotic bacteria, may lead to an imbalance in CD4^+^ T cell subsets within mesenteric lymph nodes and subsequent migration of this subset to the site of liver transplant, thereby expediting the early progression of AR. Studies have also shown that IRI after LT is associated with increased levels of IL‐17 in the portal vein plasma and the small intestine, which then affects Tregs proliferation.^331^ Following LT, dysbiosis can be identified by an increase in segmented filamentous bacteria, which contribute to the production of IL‐17, and a rise in lactobacilli. In another mouse model, elevated levels of lactobacilli were found to boost IL‐17 expression by interacting with the resident T lymphocytes in Peyer's patches.^332^, ^333^ As a result, if these same interactions take place, dysbiosis may exacerbate the IRI‐induced progression of early ACR in the human liver.

### 5.2 Prevention and treatment

Preventing infections is crucial following LT. Alterations in the immune microenvironment and the use of immunosuppressants after the transplant can further disrupt the balance of the gut–liver axis and trigger an inflammatory response in liver immune cells.^334^ Given the intricacies of immune balance, the comprehensive reduction of infection risk can be achieved through preoperative prevention and postoperative regulation. Both animal experiments and clinical studies have demonstrated that antibiotic pretreatment can enhance the production of the anti‐inflammatory prostaglandin E2 (PGE2) and increase the expression of hepatic EP4 in transplant recipients. This, in turn, reduces the count of proinflammatory macrophages and mitigates inflammatory damage.^335^ This further demonstrates that the use of antibiotics by the donor before the operation can curb the impact of intestinal flora on macrophages. In postoperative prevention and treatment, some experts suggest that probiotics and synbiotics yield more favorable results in regulating the postoperative immune microecology.^336^ 3,4‐DHPPA, a microbial metabolite, has been uncovered that it has a lessening effect on macrophage‐associated inflammation.^325^ Furthermore, changing the makeup of the intestinal microbiota can influence the inflammatory response of liver immune cells and promote improved transplant tolerance. Research has shown that enhancing one's diet can raise the levels of Bacteroides fragilis and Bacteroides thetaiotaomicron in the gut, facilitating the shift of T cells toward a more tolerant direction.^337^ Clinical trials have investigated the use of Bacillus clausii probiotics for preventing infections after LT (NCT05047406). However, a meta‐analysis that evaluated the effectiveness of synbiotics and probiotics following LT did not demonstrate any improvement in patient prognosis.^338^ At present, there is a deficiency in established clinical treatment and administration guidelines for probiotics and synbiotics, which necessitates additional clinical trials for reference. Therefore, more clinical studies are required to confirm whether probiotics can enhance the prognosis of LT patients.

In essence, the liver and intestinal flora intricately connect, sustaining immune tolerance via microbial‐derived molecules and metabolites. Disruption of this equilibrium, primarily induced by ischemia–reperfusion post‐LT and the clinical administration of immunosuppressive drugs, stands as the predominant factor contributing to posttransplant infections. Validation through additional clinical trials is imperative to establish the efficacy of probiotics in preventing posttransplantation infections. Undoubtedly, infection remains a pivotal and constant concern following LT. Beyond these primary complications, immune system dysregulation posttransplantation is intricately linked to complications in other organs and the recurrence of tumors.

## 6 OTHER COMPLICATION

Due to immune imbalance and immune responses, the occurrence of additional complications after LT is frequently underestimated. Complications like biliary tract abnormalities and tumor recurrence can be partially diagnosed through imaging detection, allowing for timely and targeted treatment.^339^ Nevertheless, the emergence of these complications is inherently linked to immune imbalance following LT.^340^, ^341^ Modulating immune cells in the liver holds the potential for therapeutic efficacy in addressing associated complications to a certain extent. This section elucidates complications stemming from immune imbalance post‐LT, outlining the involvement of immune cells in these issues and discussing their therapeutic potential.

### 6.1 Acute kidney injury

AKI is a common complication following LT, with an incidence ranging from 51 to 94%.^342^, ^343^, ^344^ Renal replacement therapy (RRT) is necessary for 15% of patients experiencing AKI.^345^ Pretransplant acute renal failure (ARF), the duration of hypoalbuminemia, dopamine therapy, LT with dysfunction levels II‐IV, reoperation, and bacterial infections all serve as risk factors for ARF after LT.^346^

Several theories exist for classifying AKI, with renal injury typically categorized based on serum creatinine (sCr) levels.^347^ While sCr and urine volume can be used to define AKI, they have limitations in predicting the early development of AKI. In cases of AKI, inflammatory macrophages have been demonstrated to release proinflammatory cytokines and migration inhibitory factor (MIF) as indicators of kidney injury severity, potentially serving as predictors for the need for RRT.^348^ In the kidney, MIF is weakly expressed in some glomerular epithelial cells and approximately half of cortical tubules.^349^ Increased MIF concentrations promote cytokine release, including IL‐6 and IL‐8.^350^ Clinically, serum MIF levels are increased in patients with chronic kidney disease, a condition that coincides with oxidative stress and endothelial activation.^351^ According to evidence‐based medical research analysis, plasma MIF levels and the change in serum creatinine (ΔsCr) offer better predictive accuracy for severe AKI development compared with using sCr alone. Moreover, plasma MIF demonstrates superior predictive ability for the need for RRT in comparison with sCr.^352^ MIF and neutrophil gelatinase‐associated lipocalin‐2 exhibit strong predictive value for AKI after LT.^353^ Even though MIF, a proinflammatory macrophage product, has not been explicitly incorporated as a predictor for diagnosing AKI after LT, clinical studies have shown its potential. There's also ongoing consideration of valproic acid, a histone deacetylase inhibitor, which has demonstrated positive effects on tissue damage and is being explored as a treatment option for post‐LT AKI (NCT04531592)^354^ (Table 2).

**TABLE 2:** Completed or active clinical trials for the treatment of IRI, AR and other complications after LT in the past 5 years.

Treatment	Clinical trial identification	Study date	Phase	Indication	Primary measure	Status
Valproic acid	NCT04531592	2020–2021	II	Liver ischemia–reperfusion injury; acute kidney injury	Improving Global Outcomes (KDIGO) stage based on serum creatinine (SCr) (time frame: within the first 48 h after study drug administration)	Withdrawn
Cytokine filtration	NCT04203004	2019–2023	Not applicable	Liver ischemia–reperfusion injury	Incidence of postreperfusion syndrome (time frame: intraoperatively, during the first 5 min after reperfusion of the liver graft)	Recruiting
Perla® cold preservation solution	NCT05194306	2021–2022	Not Applicable	Liver ischemia–reperfusion injury	Graft Function Rate (time frame: 7 days posttransplant)	Completed
Erythropoietin	NCT05325073	2022–2023	IV	Liver transplant rejection	The phenotype of peripheral blood mononuclear cells (PBMC);Tregs	Recruiting
Anti‐CD40 monoclonal antibody (CFZ533)	NCT03781414	2019– 2023	II	Liver transplant rejection	Proportion of patients with composite event (BPAR, Graft Loss or Death) over 12 months; rate of composite efficacy failure	Completed
Hypothermic oxygenated machine perfusion	NCT01745731	2021–2024	II	Liver transplant rejection	Model for early graft dysfunction score (time frame: 3 days); serum alanine transaminase (ALT) activity, international normalized ratio for prothrombin time (INR), and serum bilirubin concentration	Recruiting
Enteric‐coated mycophenolate (MPAs)	NCT05707520	2022–2023	Not applicable	Liver transplant rejection	Graft loss or reoccurrence of HCC, or neoplasm, or death	Recruiting
Bacillus clausii probiotic liquid	NCT05047406	2021–2023	II	Transplantation infection	Infection and mortality rates (time frame: up to 30 days postoperatively)	Active, not recruiting

*Data sources*: www.clinicaltrials.gov.

### 6.2 Biliary complication

Biliary complications (BCs) are more commonly observed after LDLT compared with deceased donor LT. This is mainly attributed to surgical factors like a small graft diameter and multiple bile duct openings, as well as nonsurgical factors, including immunologic reactions.^355^, ^356^ BCs continue to pose a substantial challenge in the context of LDLT, and this challenge likely arises from a combination of surgical and nonsurgical factors. Surgical factors encompass a range of anatomical features, while nonsurgical factors involve hepatic graft arterial hypoperfusion due to portal hypertension, IRI, and immune responses.^357^, ^358^, ^359^ Within the realm of surgical factors, the Roux‐en‐Y hepaticojejunostomy is a common type of anastomosis that has been effective in lowering the risk of BCs not only by means of the hepaticojejunostomy but also by reducing inflammation caused by bacterial migration.^360^ Mounting evidence suggests that damage to the biliary epithelial cell barrier leads to bile leakage, which carries intestinal PAMPs that trigger the activation of innate immune cells. This process is accompanied by the production of proinflammatory Th1 cytokines, such as IFN‐γ and TNF resulting in a biliary cirrhosis response.^361^ Research has demonstrated that the microbiota present in the biliary tract can influence BCs in conjunction with immune cells. Clostridium, for instance, has been observed to modulate the activity and cytokine production of Tregs and plays a role in maintaining colonic homeostasis.^362^ In cases of primary sclerosing cholangitis, there is an increase in Clostridium content, which subsequently triggers biliary inflammation.^363^ While initiating surgical procedures can reduce the occurrence of BCs in LT, the role of immune cells in the development of BCs should not be overlooked.

### 6.3 Recurrence of hepatocellular carcinoma

The risk of tumor recurrence remains following LT. Postoperative monitoring of tacrolimus levels and considering salvage LT in the event of recurrence can help reduce the mortality associated with recurrence.^364^, ^365^ As our understanding of tumor biology deepens, it becomes increasingly evident that the tumor immune microenvironment is intricately linked to the occurrence, progression, and recurrence of tumors.^366^ In the realm of innate immune cells, tumor‐associated macrophages (TAMs) and myeloid‐derived suppressor cells (MDSC) have been shown to have strong associations with hepatic cell carcinoma (HCC) metastasis and recurrence.^367^, ^368^ Clinical studies have confirmed that elevated levels of TAMs in the surrounding tissues of HCC patients are linked to a poorer prognosis. The underlying mechanisms involve immunosuppression, neovascularization, and resistance to treatment.^369^, ^370^ The macrophages that make up TAM primarily release lower levels of TNF and IL‐12, while exhibiting higher expression of IL‐10. These factors contribute to the inhibition of inflammation.^371^ Other pathways, such as FGF19/FGFR4, have been demonstrated to enhance the production of PD‐1 and chemokine ligand 2, consequently promoting HCC metastasis.^372^ At the genetic level, Zhou et al.^373^ conducted a gene mutation screening in human liver cancer samples, identifying mutated genes that include WNK2, RUNX1T1, CTNNB1, TSC1, and TP53. He also demonstrated that the inactivation of the WNK2 gene leads to increased infiltration of TAMs, consequently promoting liver cancer recurrence. Furthermore, clinical trials have been conducted to target the TYRO3, AXL, and MER receptors on the surface of TAMs using cabozantinib.^374^ Essentially, MDSCs are triggered by cytokines, PGE2, COX2, and the classical NF‐κB pathway.^375^ Furthermore, research has indicated that MDSCs impede the activation of immune cells through the Tim‐3/galectin‐9 pathway, which, in turn, fosters tumor recurrence.^376^ In contrast to innate immune cells, Tregs play a more significant role in the T cell system and have regulatory functions. Flow cytometry screening revealed elevated IFN‐γ secretion from Tregs in HCC patients, leading to CTL inhibition. This effect can be reversed through PD‐1 inhibitor blockade when compared with healthy individuals.^377^ To sum up, the mechanism involving immune cells in tumor recurrence among HCC patients who have undergone LT is complex, demanding additional research and targeted therapeutic approaches.

## 7 CONCLUSION

The immune system creates a complex microenvironment in the liver. Following LT, the interplay between ischemia–reperfusion and communication among immune cells and cytokines makes it challenging to achieve favorable outcomes with drug therapy alone. However, by gaining a deeper understanding of the inflammatory factors and the key effector cells involved in LT, we can more effectively identify specific cells for targeted treatment.

Macrophages play a pivotal role in initiating inflammatory and immune responses following LT. They are involved throughout the entire process of LT, including inflammation, cell damage, and the development of late immune tolerance. In subsequent reactions, T cells, as essential effector cells, collaborate with macrophages to engage in both injury and repair processes. Even though we have not yet fully comprehended the surface characteristics and mechanisms of effector cells, the emergence of single‐cell sequencing, spatial transcriptome analysis, and nanopore sequencing provides optimism for the discovery of new targets of action. Simultaneously, the composition of the infusion remains an area of exploration with substantial potential. It has been observed that including active components for clearing ROS and inhibitory cytokines in perfusion can enhance the immune microenvironment. Nevertheless, current regulatory perfusion components are predominantly single‐agent. Investigating the incorporation of multiple effective drugs can open up new avenues for future clinical research.

LT involves two primary processes, IRI and immune rejection, both of which may lead to subsequent complications. The immune damage to the liver following transplantation can be partially alleviated through the utilization of advanced perfusion techniques and perfusion fluids. Presently, the primary emphasis in donor liver perfusion is on developing newer and more effective perfusion techniques. Modifying the temperature of the perfusion fluid and employing mechanical perfusion can effectively mitigate posttransplantation injuries to a certain degree. Therefore, the further development of more efficient perfusion devices could serve as a promising step in enhancing the outcomes of LT and reducing complications. In the later stages of LT, immune tolerance functions represented by macrophages and T cells have become apparent. Clinical infusion of DCregs, Tregs, and NK cells has been conducted to suppress posttransplant rejection and prevent HCC recurrence. Nevertheless, the safety and preparation methods for tolerogenic cell infusion therapy require further exploration and enhancement. Mregs, a recently discovered regulatory immune cell in the realm of organ transplantation, have not been thoroughly explored regarding their role and effectiveness in LT. Therefore, with the aid of emerging technologies, optimizing donor liver preservation to mitigate posttransplant immunity, alongside the development of new drugs and therapies in the later stages, can more effectively decrease complications following LT and enhance patient prognosis.

## AUTHOR CONTRIBUTION

X. X. and R. G. C. completed the concept and key design of the study. Y. C. H., X. N. X., and S. P. collected literature. G. R. C., Y. C. H., and X. H. wrote a draft revision of the original. All authors have read and approved the article.

## CONFLICT OF INTEREST STATEMENT

The authors declare no conflict of interest.

## ETHICS STATEMENT

Not applicable.

## Data Availability

Not applicable.
